# TRAF-STOP alleviates osteoclastogenesis in periodontitis

**DOI:** 10.3389/fphar.2023.1119847

**Published:** 2023-05-15

**Authors:** Yaxian Huang, Jinyan Wu, Chi Zhan, Rong Liu, Zhaocai Zhou, Xin Huang, Yaguang Tian, Zhengmei Lin, Zhi Song

**Affiliations:** ^1^ Hospital of Stomatology, Guangdong Provincial Key Laboratory of Stomatology, Guanghua School of Stomatology, Sun Yat-sen University, Guangzhou 510055, Guangzhou, China; ^2^ Yunnan Key Laboratory of Stomatology, Kunming Medical University School and Hospital of Stomatology, Kunming 650106, China; ^3^ School of Medicine, South China University of Technology, Guangzhou 510006, Guangzhou, China; ^4^ Department of Stomatology, Hainan General Hospital, Hainan Affiliated Hospital of Hainan Medical University, Haikou 570311, Hainan, China.

**Keywords:** periodontitis, osteoclast, CD40L-CD40-TRAF6 axis, TRAF-STOP, NF-κB

## Abstract

The enhanced osteoclastogenesis contributes to alveolar bone resorption in periodontitis, which increases the risk of tooth loss. To reduce bone destruction, the inhibition of osteoclast development is proposed as a feasible treatment. CD40L-CD40-TRAF6 signal transduction plays a crucial role in inflammation, but how it regulates osteoclast activity in periodontitis has not been elucidated. In this study, we showed the potential role of CD40L-CD40-TRAF6 signaling in periodontitis. CD40L obviously promoted osteoclast formation and bone resorption capacity *in vitro*. Mechanistically, we found that osteoclastogenesis was enhanced by the overexpression of NFATc1 and NF-κB activation. Importantly, osteoclast activity was effectively suppressed by TRAF-STOP, a small molecular inhibitor of TRAF6. Furthermore, local injection of TRAF-STOP-loaded injectable PLGA-PEG-PLGA hydrogel could alleviate ligation-induced periodontitis *in vivo*. Taken together, TRAF-STOP shows promising clinical efficacy in periodontitis through alleviating osteoclastogenesis.

## 1 Introduction

Periodontitis is a chronic inflammatory disease caused by pathogenic microflora, contributing to the continuous destruction of the periodontium including gingiva, periodontal ligament, and alveolar bone (Hajishengallis, 2015). As the sixth most prevalent condition worldwide, periodontitis is a major public health problem requiring high attention (Chapple, 2014; Kassebaum et al., 2014). Moreover, clinical and epidemiological evidence demonstrates that periodontitis shows a close relationship with rheumatoid arthritis, atherosclerosis, and cardiovascular disease (Hajishengallis, 2015). Though local mechanical therapies like scaling and root planning have been the conventional treatment for periodontitis, effective adjuvant drugs are still under study (Glickman, 1971).

Excessive bone resorption by osteoclasts is regarded as one of the major factors leading to alveolar bone loss, which is a significant hallmark of periodontitis (Jiao et al., 2014; Hathaway-Schrader and Novince, 2021). Bone homeostasis depends on the balance between osteoblast-mediated bone formation and osteoclast-mediated bone resorption (Wei et al., 2010). Though the critical role of NF-κB signaling in osteoclast maturation has been studied extensively (Iotsova et al., 1997; Arron and Choi, 2000), the molecular mechanisms of NF-κB signaling in osteoclast linked to periodontitis remain unclear.

The costimulatory CD40L-CD40 dyad plays a pivotal role in inflammatory processes and autoimmune diseases such as rheumatoid arthritis, atherosclerosis, and cardiovascular disease (Peters et al., 2009; Strohm et al., 2022). The binding of CD40L inspires CD40 to recruit adaptor proteins, the tumor necrosis factor receptor-associated factors (TRAFs), resulting in the activation of the corresponding downstream signal (Lim, 2018). The only possession of the TRAF-C domain among the TRAF family doomed TRAF6 to a unique biological function. Gao et al. demonstrated that T cells promoted PTH-induced bone resorption through CD40L signaling, suggesting the activation of the CD40L-CD40 axis is beneficial to osteoclast activity (Gao et al., 2008). In addition, enhanced CD40 signals can initiate osteoclast differentiation (Kadono et al., 2005). TRAF6 plays a critical role in osteoclasts activity. The defect in osteoclast was detected in the TRAF6-deficency mice, indicating the novel role of TRAF6 in bone metabolism (Lomaga et al., 1999). Moreover, TRAF6 can mediate RANK-dependent signaling, cytoskeletal rearrangement and the expression of transcription factor NFATc1, all of which are necessary for osteoclastogenesis (Negishi-Koga and Takayanagi, 2009a; Walsh et al., 2015). Thus, we wondered whether the CD40L-CD40-TRAF6 axis would affect osteoclast activity.

TRAF-STOP is a small molecule inhibitor (SMI) specific to the CD40-TRAF6 axis but not the CD40-TRAF2/3/5 pathway (Zarzycka et al., 2015). Up to now, TRAF-STOP has achieved a satisfactory therapeutic effect in reducing atherosclerosis and neuroinflammation (Aarts et al., 2017; Seijkens et al., 2018). Importantly, precise targeting of the CD40-TRAF6 axis by TRAF-STOP treatment prevents the occurrence of thromboembolic events and/or immunosuppression compared with CD40L antagonists which may affect the other CD40-TRAFs interaction (Engel et al., 2009; Tang et al., 2021).

Poly (lactide-co-glycolide acid) and polyethylene glycol triblock copolymer (PLGA-PEG-PLGA) has been frequently utilized as drug carrier on account of their superior biocompatibility, manageable rate of biodegradation, and efficient drug administration (Gao et al., 2020). This thermosensitive hydrogel can distribute its contents via injection under free-flowing sol conditions (Correa et al., 2021). Related studies have been performed to exhibit sustained release capacities of the injectable PLGA-PEG-PLGA hydrogels containing hydrophobic drugs, shRNA, vaccines, and so on (Ma et al., 2014; Wang et al., 2017; Gao et al., 2020). The hydrogel can continuously release the drug locally, which can reduce topical administration and increase patient comfort (Russo and Villa, 2019). Besides, the improved drug bioavailability leads to better therapeutic effect in periodontitis (Wei et al., 2021). However, the efficacy of TRAF-STOP-loaded PLGA-PEG-PLGA hydrogel in the treatment of periodontitis has not been fully investigated.

In general, this study seeks to ascertain the effect of the CD40L-CD40-TRAF6 axis on osteoclastogeneis and the putative effect of TRAF-STOP in osteoclasts. In particular, we evaluated the potential treatment capacity of TRAF -STOP-loaded injectable hydrogel in periodontitis.

## 2 Methods and materials

### 2.1 Human gingiva collection

Gingival tissue of healthy individuals and patients with periodontitis was collected surgically. The healthy gingival tissue was extracted surgically from the third molars of healthy donors while gingival tissue of periodontitis was obtained from the inner wall of the periodontal pocket around the tooth which needs to be extracted because of severe periodontitis. Specifically, the healthy group should have no bleeding on probing (BOP) positive sites and show a probing depth (PD) < 3 mm. The severe periodontitis has the following features: probing depth (PD) > 6 mm、the attachment loss (AL) >5 mm and the bone absorption exceeds 1/2 of the root length. Informed consent was obtained from all donors. The study was approved by the Institutional Ethics Committee Board of the Guanghua School of Stomatology, Sun Yat-sen University (GHKQ-202211-K07-01).

### 2.2 Cell culture

Bone marrow-derived monocytes/macrophages (BMMs) were obtained from the tibia and femurs of 6-week-old male C57BL/6 mice (Animal Center of Sun Yat-sen University). Cells were seeded into 96‐well plates at a density of 1 × 10^4^/well, or 6‐well plates at a density of 5 × 10^5^/well in α-MEM (Gibco, Grand Island, NY, United States) containing 30 ng/mL M-CSF (Sino Biological Inc, Beijing, China) and 50 ng/ml RANKL (R&D Systems, Minneapolis, MN, United States) without or with CD40L (R&D Systems, Minneapolis, MN, United States) and TRAF-STOP (MedChemExpress, Shanghai, China). Cells treated with only M-CSF were used as controls. All cells were incubated in a 37°C incubator under an atmosphere with 5% CO_2_.

### 2.3 TRAP staining

Upon observation of multi-nucleated osteoclasts at 5-6 days of culture, TRAP staining was carried out to detect osteoclasts following the manufacturer’s instructions (TRAP staining kit, Sigma, MO, United States). TRAP‐positive cells with at least three nuclei are regarded as osteoclasts (Huang et al., 2022). TRAP-positive multinucleated cells (MNCs) were observed under the inverted microscope (Olympus IX71, Olympus, America) and counted by ImageJ (National Institutes of Health, Bethesda, MD, US)

### 2.4 Podosome belt immunofluorescence assay

BMMs were seeded in a 96-well plate as described above. After BMMs were differentiated into osteoclasts for 5, 6 days, cells were fixed in the 96-well plate with 4% PFA for 20 min at 37°C, followed by the permeabilization with 0.1% Triton X-100 in PBS for 10 min. After being blocked with 2% BSA for 20 min, cells were stained with Alexa Fluor 488 phalloidin (Beyotime, Shanghai, China) for 1 h at room temperature and then counterstained with DAPI for 10 min in the dark. Images of osteoclastic rings were visualized by fluorescence microscopy (Olympus IX71, Olympus, and America).

### 2.5 Bone absorption assay

BMMs were seeded at a density of 1 × 10^4^/well onto bovine bone slices in a 96-well plate with three replicates for 8, 9 days. Later, the slices were harvested and brushed thoroughly to remove the cells after the formation of mature osteoclasts. The resorption pits on slices were observed and captured by the scanning electron microscope (Merlin Compact, Zeiss, and Germany) and the area of bone resorption was quantified by ImageJ software.

### 2.6 RNA extraction, reverse transcription, and RT-qPCR

Total RNA was extracted from periodontal tissue and cells with the RNA-Quick purification kit (Yishan, Shanghai, and China) based on the instructions. Sample concentrations were determined using a NanoDrop ND-1000 spectrophotometer (Thermo Scientific, MA, and United States). cDNA synthesis was carried out using a PrimeScript™ RT Master Mix Kit (TaKaRa, Kyoto, Japan). qRT-PCR was performed using a Light Cycler 96 instrument (Roche, Basel, Switzerland) with SYBR Green I Master Mix (Roche, Basel, Switzerland). All target genes were normalized to the expression of GAPDH mRNA. The specific primers used for detecting mRNA are listed in Supplementary Table S1.

### 2.7 Western blot analysis

Protein from cells and tissues was extracted using radioimmunoprecipitation assay (RIPA) buffer (Millipore, MA, and United States) with protease and phosphatase inhibitors (Cwbio, Beijing, and China) on ice for 30 min. The nuclear fraction was acquired through nuclear and cytoplasmic protein extraction kit (Beyotime, Shanghai, and China) according to the manufacturer’s instruction. Ultrasonic homogenizers were used to disrupt the RIPA buffer-extracted lysates. Later, the supernatants were harvested by centrifugation for 25 min at 12000 rpm and their concentration was measured by a BCA protein assay kit (Cwbio, Beijing, and China). 4%–20% SDS-PAGE gel (SurePAGE™, GenScript, Nanjing, and China) was used to separate the proteins, which were then transferred to a polyvinylidene fluoride (PVDF) membrane (Millipore, MA, and United States). Blocking was performed with 5% BSA in TBST for 1 h, followed by incubation with indicated primary antibodies at 4°C overnight and secondary antibodies for 1 h at room temperature with mild shaking. Protein bands were visualized using the ECL system (ZEN-BIOSCIENCE, Chengdu, and China) and all images were analyzed with ImageJ software. The antibody information is listed in Supplementary Table S2.

### 2.8 Cell viability assay

Cell Counting Kit-8 (CCK-8) test was carried out to assess the effect of TRAF -STOP on cell viability. Briefly, BMMs were seeded in 96-well plates at a density of 1 × 10^4^/well in triplicate. After 24 h of incubation, various TRAF-STOP concentrations (0–20 µM) were added to BMM cells for 48 h. Each well was incubated for an additional 2 h with CCK-8 solution (Dojindo Molecular Technology, Kumamoto, Japan) at 37°C under 5% CO_2_ and humidified atmosphere. Cell viability was determined by a microplate reader (BioTek, Vermont, United States) at 450 nm.

### 2.9 Polymer synthesis and characterization

PLGA-PEG-PLGA triblock copolymers were synthesized through ring-opening copolymerization of D, L-lactide (D, L-LA), and glycolide (GA) with Poly (ethylene glycol) (PEG) as the macroinitiator and Sn (Oct)2 as the catalyst. PEG was purchased from Sigma. D, L-LA, and GA were purchased from Aladdin Reagent (Shanghai) Co., LTD. Later, the crude products were dissolved in cold water (4°C–8°C), and the polymer solution was heated to 80°C to precipitate the polymer. The precipitated polymer was separated from the supernatant by pouring. And the product was freeze-dried.

The composition and structure of the copolymers were examined by 1H NMR spectrum, which was performed on a 400 MHz Bruker spectrometer using CDCl3 as a solvent. Molecular weights (MW) of the copolymer were evaluated by gel permeation chromatography (GPC, Waters) with tetrahydrofuran as eluting solvent at a flow rate of 1.0 mL/min. The MW was calibrated with polystyrene standard. Additionally, the inverted vial technique was used to investigate the sol-gel phase transition. PLGA-PEG-PLGA polymer structure has a certain proportion of hydrophilic and hydrophobic groups. At low temperature, the hydrophilic groups and water molecules dissolve in water due to the strong carbon-oxygen hydrogen bonding force between PLGA-PEG-PLGA molecular chains, at which time PLGA-PEG-PLGA polymer is in solution state. With the increase of temperature, some of the hydrogen bonding forces gradually weakened, while the hydrophobic forces in the PLGA-PEG-PLGA polymer chains continuously increased. When reaching a certain temperature, the polymer chains aggregated with each other and a bulk phase transition occurred, and the PLGA-PEG-PLGA polymer was in the gel state. Therefore, we examined whether the PLGA-PEG-PLGA polymer is temperature-sensitive by the inverted tube method.

### 2.10 Animals

All animals used in this study were male C57BL/6J mice aged 6–8 weeks purchased from Sun Yat-sen University (Guangzhou, China). The animal experiments were approved by the Ethics Committee of Sun Yat-sen University (Document No. 2021001578). Mice were randomly and equally divided into three groups (n = 5) as follows: (a) PBS injection; (b) TRAF-STOP injection; (c) TRAF-STOP + hydrogel injection. PBS was used as vehicle control. 5-0 thread was ligated at the submarginal position around the bilateral maxillary second molars and kept for 10 days to induce periodontitis (Abe and Hajishengallis, 2013; Marchesan et al., 2018). For injection of medication, medical sterile insulin syringe (320312) from BD was utilized. The insulin needle is suited for local injection because it is smaller with a diameter of 0.33 mm. Drugs were injected into the central palatal gingiva of the maxillary second molar after ligature removal every 3 days for 1 month.

### 2.11 Micro-CT analysis

Maxillae from experimental mice were collected and scanned with high-resolution micro-CT (Scano Micro-CT, μCT50, Switzerland). Image acquisition was performed at 70 kV, 114 mA, and 10-um increments. RadiAnt DICOM Viewer (Medixant, Poznan, Poland) was used to reconstruct three-dimensional images and measure the distance of the cementoenamel junction to the alveolar bone crest (CEJ-ABC distance) for the evaluation of bone loss.

### 2.12 Histology, immunohistochemistry analysis, and immunofluorescence staining

Both the collected human gingiva and the maxillae were fixed in 4% paraformaldehyde (PFA) for 24 h and the latter were decalcified in 0.5 M EDTA for 4 weeks. After being embedded in paraffin, all samples were sectioned to 4–5 μm, followed by hematoxylin and eosin (H&E), tartrate-resistant acid phosphatase (TRAP) staining, immunohistochemistry (IHC) staining as well as immunofluorescence (IF) staining. H&E staining was used to assess bone loss by showing the distance between the cementoenamel junction and alveolar bone crest (CEJ-ABC distance). The TRAP staining was performed using a commercial TRAP kit (TRAP staining kit, Sigma, MO, United States). TRAP-positive cells are stained as dark purple multinucleated cells located on the bone perimeter within a resorption lacuna. Immunohistochemistry (IHC) staining was performed with DAB Detection Kit (Polymer) kit (Gene Tech, Shanghai, China) according to the manufacturer’s instructions. The primary antibodies used were the NFATc1 antibody (1:100, A1539, ABclonal, Wuhan, China), CD40 (1:100, A0218, ABclonal, Wuhan, China), TRAF6 (1:100, A0973, ABclonal, Wuhan, China). After removal of the secondary antibody, slides were dehydrated, hematoxylin immersed, differentiated, and mounted with neutral gum and image. As for IF staining, sections were counterstained with DAPI at room temperature for 5 min and samples were visualized using a confocal laser scanning microscope (LSM780, Carl Zeiss Microscopy, Oberkochen, Germany).

### 2.13 Statistical analysis

All data are indicated as the mean ± standard deviation. Student’s t-tests were used for the comparison of two groups while one-way ANOVA was used for the comparison of three or more groups. *p* < 0.05 was considered statistically significant. All statistical analyses were performed using GraphPad Prism.

## 3 Results

### 3.1 Elevated expression of CD40 and TRAF6 in periodontitis

**FIGURE 1:**
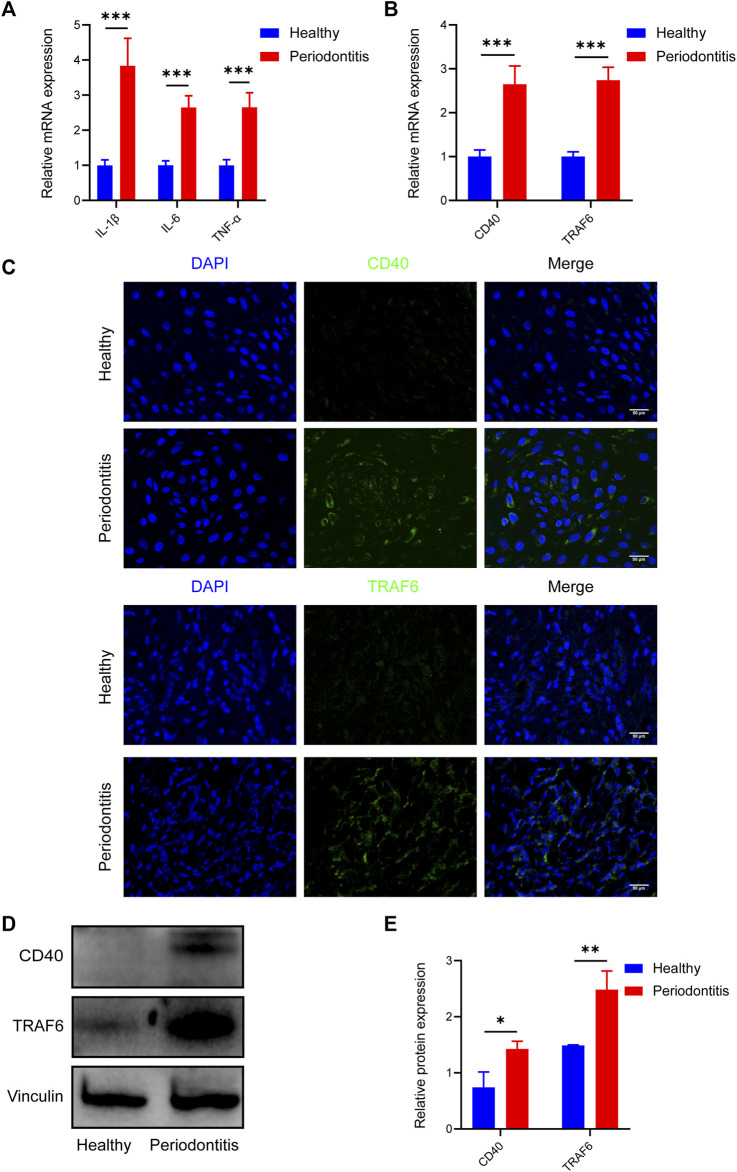
Elevated expression of CD40 and TRAF6 in periodontitis patients **(A)** The mRNA levels of IL-1β, IL-6 and TNF-α in the gingiva of healthy individuals (*n* = 6) and patients with periodontitis (*n* = 6) were analyzed by RT-qPCR. All target genes were normalized to the expression of GAPDH mRNA. **(B)** The mRNA levels of CD40 and TRAF6 in the gingiva of healthy individuals (*n* = 6) and patients with periodontitis (*n* = 6) were analyzed by RT-qPCR. All target genes were normalized to the expression of GAPDH mRNA. **(C)** Immunostaining fluorescence for CD40 and TRAF6 in the gingiva of each group. Nuclei were stained with DAPI. Scale bar = 50 μm. **(D)** Representative Western blot showing protein levels of CD40 and TRAF6. **(E)** Statistical analysis of Western blot data to determine the relative intensity of CD40 and TRAF6. (Data are expressed as mean ± SD. *p < 0.05, **p < 0.01, ***p < 0.001.).

To investigate the variable expression of CD40 and TRAF6 in healthy individuals and patients with periodontitis, we harvested the gingival samples separately. Initially, to ensure the reliability of the samples, inflammatory cytokines including IL-1β, IL-6, and TNF-α of each group were evaluated by RT-qPCR, all of which are significantly upregulated in periodontitis (Figure 1A). Meanwhile, RT-qPCR data revealed approximately threefold higher CD40 and TRAF6 gene expression in periodontitis compared with the healthy group (Figure 1B). Consistently, immunofluorescence staining also exhibited higher expression of CD40 and TRAF6 in the gingiva with periodontitis (Figure 1C). Western blotting results further confirmed the upregulation of CD40 and TRAF6, both of which showed a nearly 1.5-fold increase in the periodontitis group (Figures 1D, E). Taken together, these data indicated that the CD40-TRAF6 signaling pathway may play a significant role in periodontitis.

### 3.2 TRAF-STOP suppresses CD40L- and RANKL-induced osteoclast differentiation and inhibits NF-κB signaling *in vitro*

**FIGURE 2:**
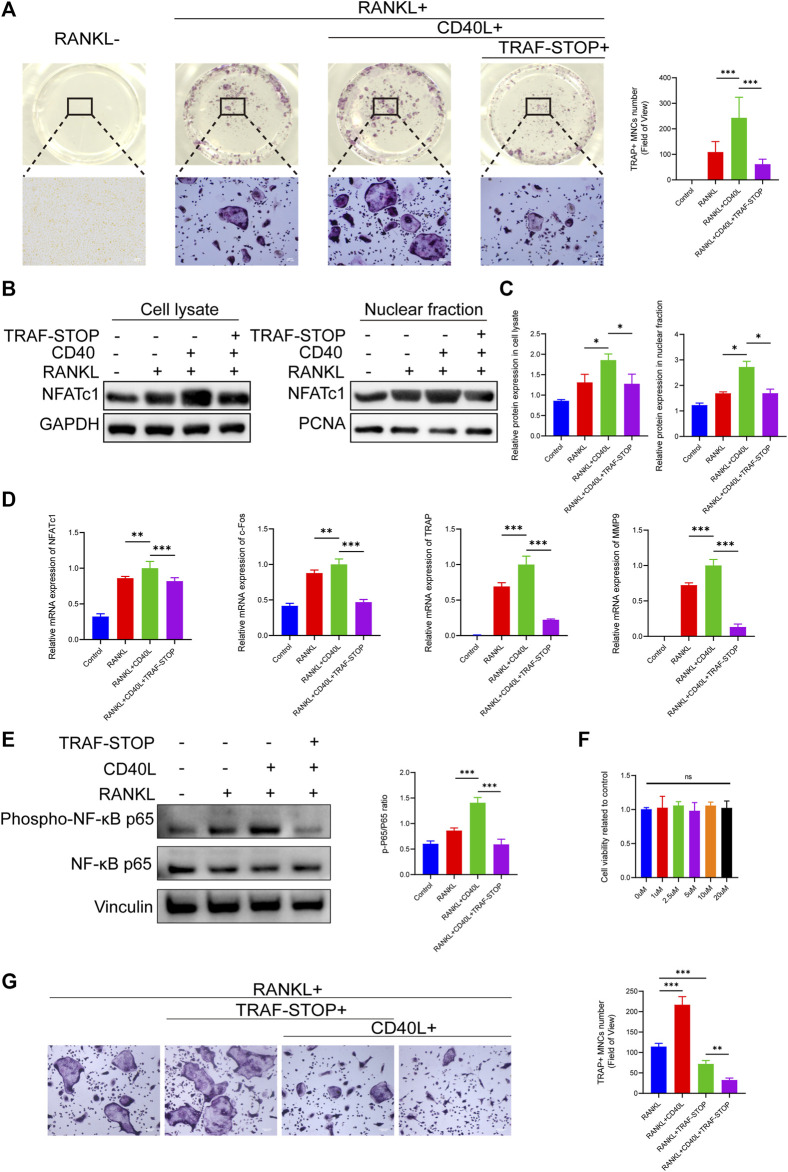
TRAF-STOP suppresses RANKL-induced osteoclast differentiation *in vitro* **(A)** Representative images of TRAP-stained multinucleated osteoclasts treated with CD40L (20 ng/ml) in the absence or presence of TRAF-STOP (*n* = 5). Scale bar = 100 μm. TRAP-positive multinucleated (nuclei > 3) cells (TRAP + MNCs) were quantified. **(B)** The NFATc1 protein expression in cell lysate and nuclear fraction were presented by Western blotting (*n* = 5). **(C)** Quantitative analysis of NFATc1 normalized to GAPDH/PCNA expression. **(D)** Osteoclast-specific genes NFATc1, c-Fos, TRAP, and MMP9 were analyzed using quantitative PCR. All target genes were normalized to the expression of GAPDH mRNA (*n* = 5). **(E)** The total and phosphorylated P65 protein expressions were measured by Western blotting. Relative protein expression of p-P65/P65 was quantified by ImageJ software (*n* = 5). **(F)** Effect of TRAF-STOP on BMMs’ viability and proliferation was assessed by CCK8 assay at 48 h with indicated concentrations (*n* = 5). **(G)** Representative images and statistical analysis of TRAP-stained multinucleated osteoclasts treated with or without TRAF-STOP in the absence or presence of CD40L (*n* = 5). Scale bar = 100 μm. (Data are expressed as mean ± SD. *p < 0.05, **p < 0.01, ***p < 0.001.).

To investigate the effects of TRAF-STOP on osteoclastogenesis, BMMs in a medium containing RANKL and M-CSF were exposed to CD40L in the presence and absence of TRAF-STOP. TRAP staining exhibited that CD40L successfully promoted the differentiation of osteoclasts, as indicated by the increase in the number and size of TRAP + cells (Figure 2A). However, the addition of TRAF-STOP attenuated osteoclast formation. The number of TRAP-positive cells declined from 243.1/well (without TRAF-STOP) to 61.6/well (with 1 µM TRAF-STOP). Moreover, both the expression of NFATc1 in cell lysate and nuclear fraction detected by Western blot were consistent with the results above (Figures 2B, C). Similarly, RT-qPCR data revealed the striking upregulation of osteoclast-specific marker genes expression including NFATc1, c-Fos, TRAP, and MMP9 after the treatment of CD40L, which was reversed by TRAF-STOP (Figure 2D). In the meantime, we found that CD40L treatment dramatically increased NF-κB activation (the phosphorylation of P65) with a percentage of seventy by Western blotting, whereas TRAF-STOP treatment resulted in a 60% decrease in NF-κB activation (Figure 2E). Furthermore, the CCK8 assay showed TRAF-STOP had no cytotoxic effects on BMMs at concentrations below 20uM (Figure 2F). Interestingly, we found that TRAF-STOP could inhibit RANKL-induced osteoclast development in the absence of CD40L, and this impact was more pronounced in the group including CD40L as well (Figure 2G). These data suggest that TRAF-STOP treatment in BMMs inhibits NFATc1 expression and NF-κB activation, which reduces osteoclast formation.

### 3.3 TRAF-STOP attenuates CD40L- and RANKL-induced osteoclastic bone resorption capacity

**FIGURE 3:**
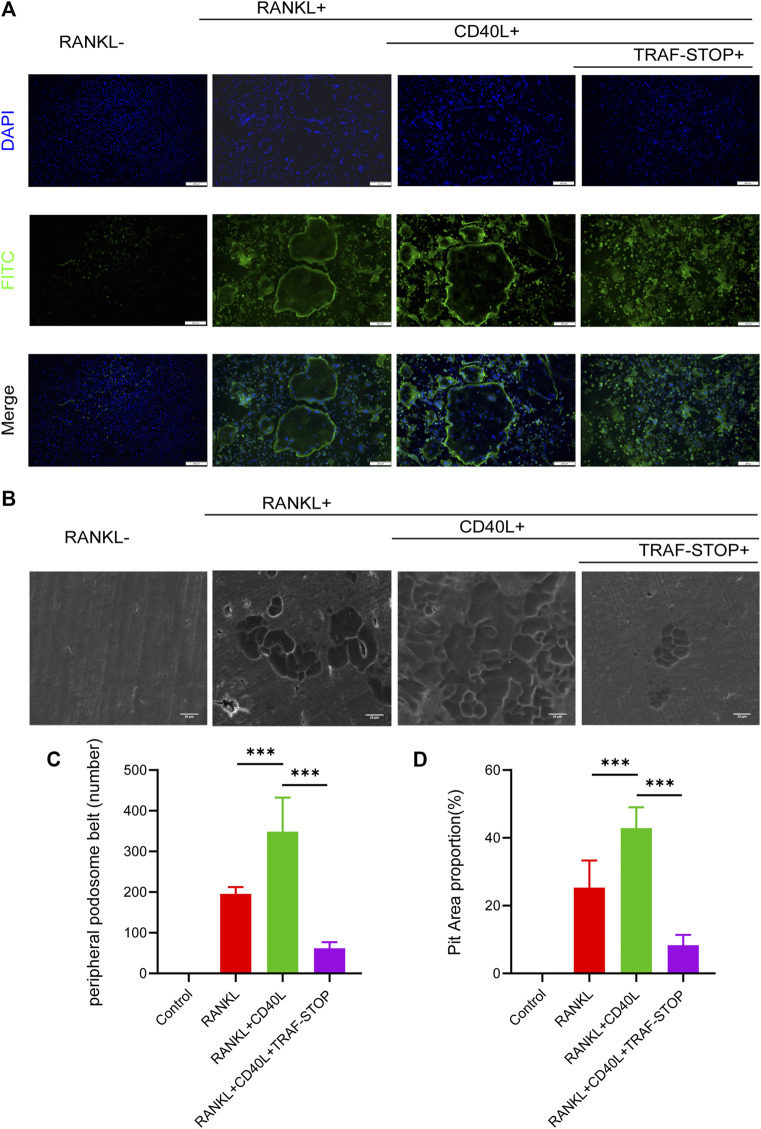
TRAF-STOP attenuates RANKL-induced osteoclastic bone resorption capacity **(A)** Representative images of podosome belts treated with CD40L in the absence or presence of TRAF-STOP (*n* =5). Nuclei were stained with DAPI. Scale bar = 200 μm. **(B)** Representative images of bovine slices resorption treated by the CD40L or TRAF-STOP (*n* = 5). Scale bar = 20 μm. **(C)** Statistical analysis of the number of podosome belts using ImageJ. **(D)** Statistical analysis of the bone resorption area in each group was quantified by ImageJ. (Data are expressed as mean ± SD. *p < 0.05, **p < 0.01, ***p < 0.001.).

A well-formed podosome actin belt was regarded as the mature osteoclast actin cytoskeleton, reflecting osteoclastic bone resorption capacity (Leightner et al., 2020). Thus, immunofluorescence staining of mature osteoclasts was carried out to evaluate the impact of CD40L and TRAF-STOP on the function of the osteoclast (Figures 3A, C). CD40L stimulated the larger and clearer actin belt compared to RANKL-only treated controls. Interestingly, the size and amount of actin ring were decreased under the treatment of TRAF-STOP and only cluster-like organization was detected. As for the pit formation assay (Figures 3B, D), the treatment of CD40L led to 1.7-fold more resorption pits, while TRAF-STOP reduced pit resorption on bovine slices caused by CD40L and RANKL-stimulated osteoclasts, further confirming the inhibitory effect of TRAF-STOP on bone resorption. Collectively, these results indicate TRAF-STOP represses the bone resorption of osteoclasts *in vitro*.

### 3.4 Characterization of PLGA-PEG-PLGA hydrogel

**FIGURE 4:**
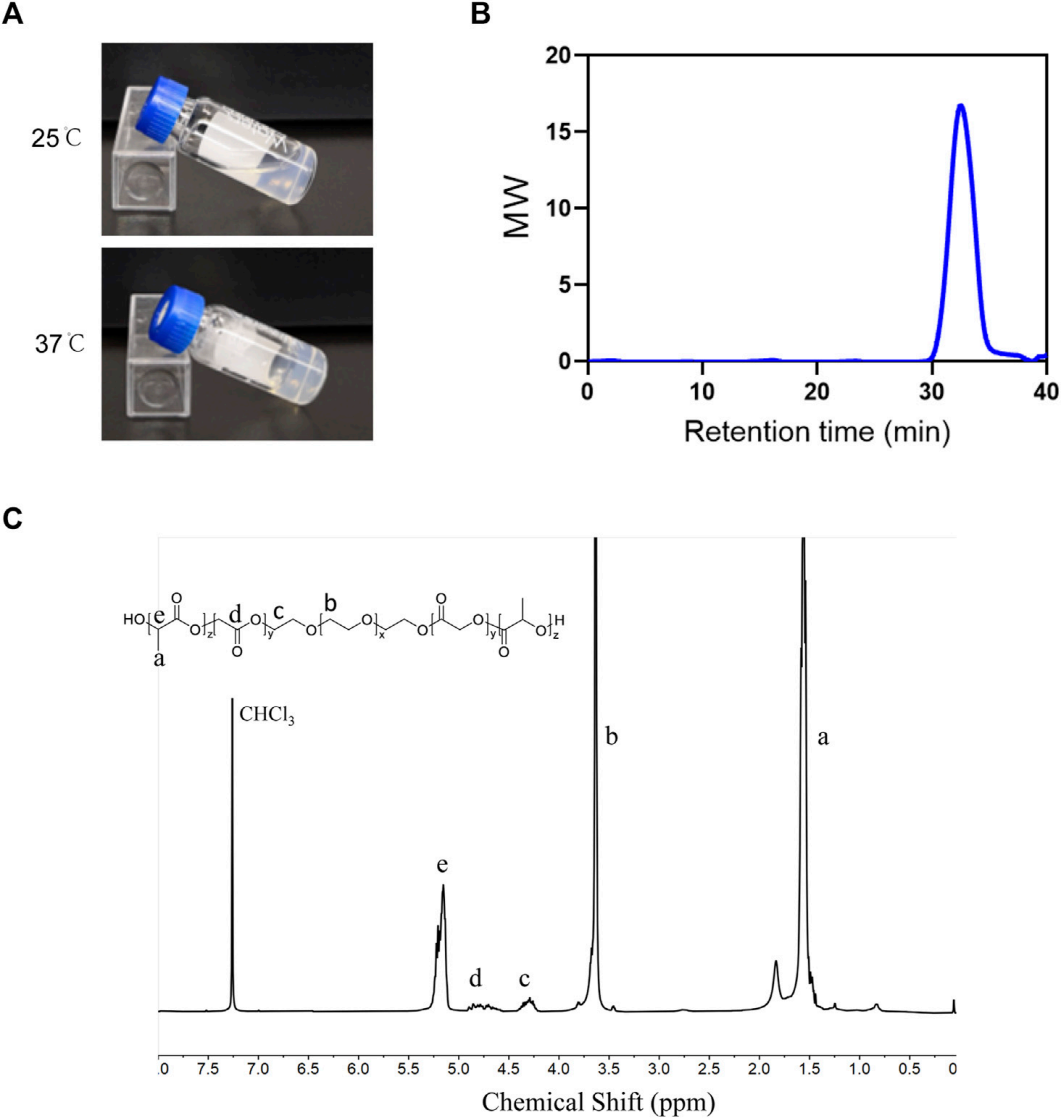
Characterization of PLGA-PEG-PLGA hydrogel **(A)** Photographs of the PLGA–PEG–PLGA formulation at indicated temperatures. **(B)** GPC traces of the PLGA-PEG-PLGA. **(C)** The ^1^H-NMR spectrum of the PLGA-PEG-PLGA triblock copolymer (400 MHz, CDCl_3_).

The PLGA-PEG-PLGA triblock copolymer was synthesized as mentioned above. PLGA-PEG-PLGA hydrogel was in a liquid state at 25°C and converted into non-flowing hydrogels upon exposure to the incubator at 37°C (Figure 4A). The ^1^H NMR spectra and GPC were used to establish the copolymer’s chemical composition and molecular weight. As demonstrated in Figure 4C, the ^1^H NMR spectrum exhibited characteristic proton signal peaks of the copolymer, and the signals appearing at 5.20 and 4.80 ppm showed LA/GA molar ratio was 13/1. The polymer dispersity index (PDI) determined by GPC read 1.17, and the average MW value of the synthesized copolymer was 6640 (Figure 4B), indicating the sample was successfully synthesized as desired.

### 3.5 Local administration of TRAF-STOP/hydrogel attenuates alveolar bone loss and periodontal inflammation in periodontitis

**FIGURE 5:**
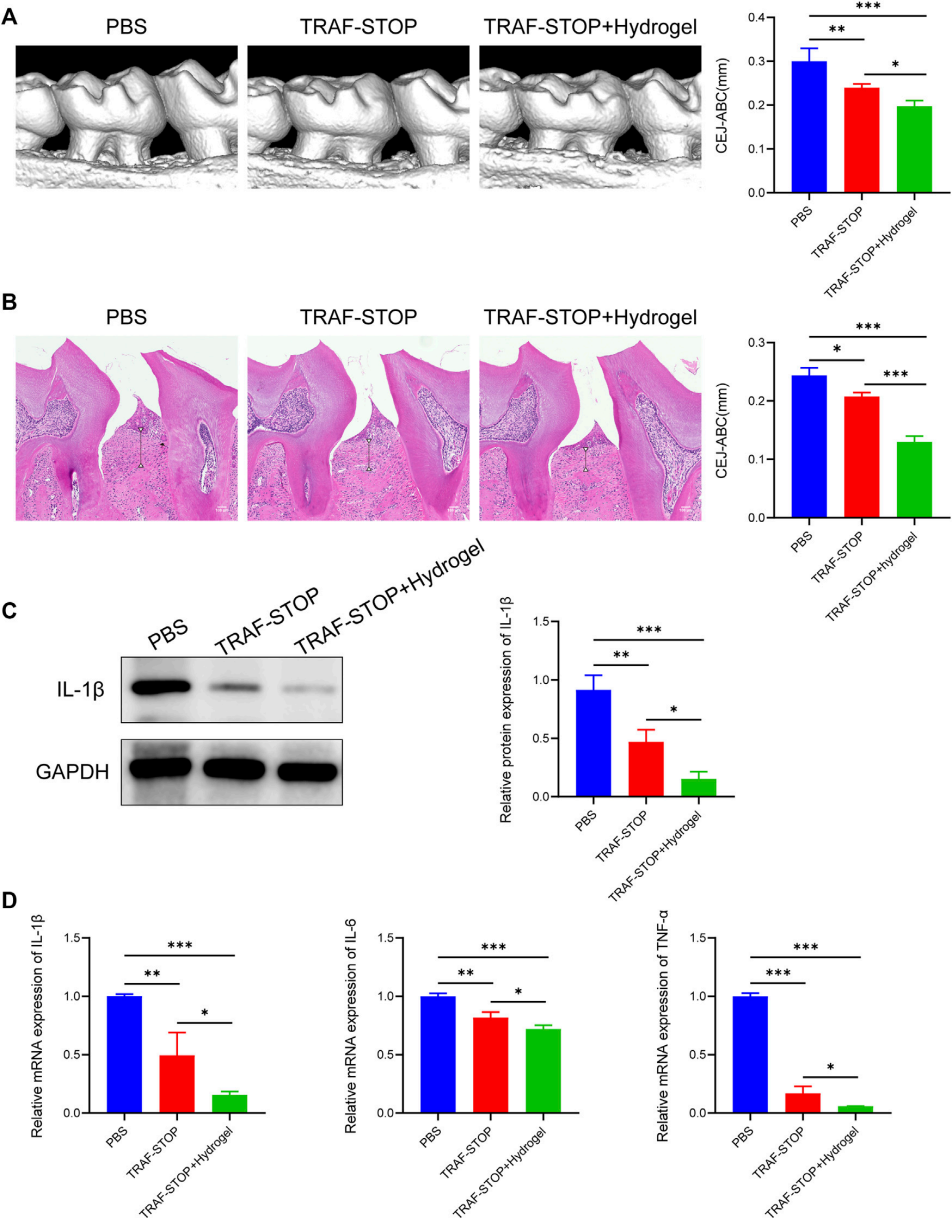
Local administration of TRAF-STOP/hydrogel attenuates alveolar bone loss and periodontal inflammation in periodontitis **(A)** Representative images of 3D reconstructions of maxillae from each group and statistical analysis of the Micro-CT-based CEJ-ABC distance (*n* = 5). The vertical line indicates the distance between CEJ-ABC. **(B)** Representative histological images of H&E -stained maxillae sections in various groups and statistical analysis of the H&E staining-based CEJ-ABC distance (*n* = 5). The vertical line exhibits the distance between CEJ to ABC. Black arrow: the multinucleated giant cell. Scale bar = 100 μm. **(C)** The IL-1β protein expression levels of each group were presented by Western blotting (*n* = 5). Quantitative analysis of IL-1β normalized to GAPDH expression. **(D)** Inflammation-related genes IL-1β,IL-6 and TNF-α were analyzed by quantitative PCR. All target genes were normalized to the expression of GAPDH mRNA. (Data are expressed as mean ± SD. *p < 0.05, **p < 0.01, ***p < 0.001.).

The prospective role of TRAF-STOP in the progression of periodontitis *in vivo* was investigated in the ligature-induced periodontitis model. Distance between CEJ (cement–enamel junction) and ABC (alveolar bone crest) was used to evaluate the degree of bone resorption (Xin et al., 2022). The CEJ-ABC distance was found to be nearly 2-fold higher in the periodontitis group compared to the control group (Supplementary Figure S1), showing that our periodontitis model was successfully established. Micro-CT-based quantitative analyses of the alveolar bone indicated that TRAF-STOP partially alleviated ligature-induced bone resorption and the TRAF-STOP/hydrogel-treated group exhibited an approximately 1.5-fold decrease in distance compared to the PBS group (Figure 5A). Additionally, H&E staining of the PBS-injection group exhibited inflammatory cell infiltration, and severe bone loss, all of which were the manifestations of alveolar bone resorption (Figure 5B). Moreover, the multinucleated giant cell was detected in PBS group, which was recognized as the hallmark of chronic inflammation (McNally and Anderson, 2011). However, this situation was dramatically attenuated by TRAF-STOP, especially in the TRAF-STOP/hydrogel-treated group. Moreover, IL-1β in periodontal tissue was shown the lowest expression level in the TRAF-STOP/hydrogel treatment group (Figure 5C), which was consistent with the mRNA levels of IL-1β, IL-6, and TNF-α (Figure 5D), suggesting the combination of TRAF-STOP and hydrogel might be more effective than TRAF-STOP alone for the treatment of periodontitis.

### 3.6 Local administration of TRAF-STOP/hydrogel alleviates osteoclast activity via suppression of the NF-κB signaling

**FIGURE 6:**
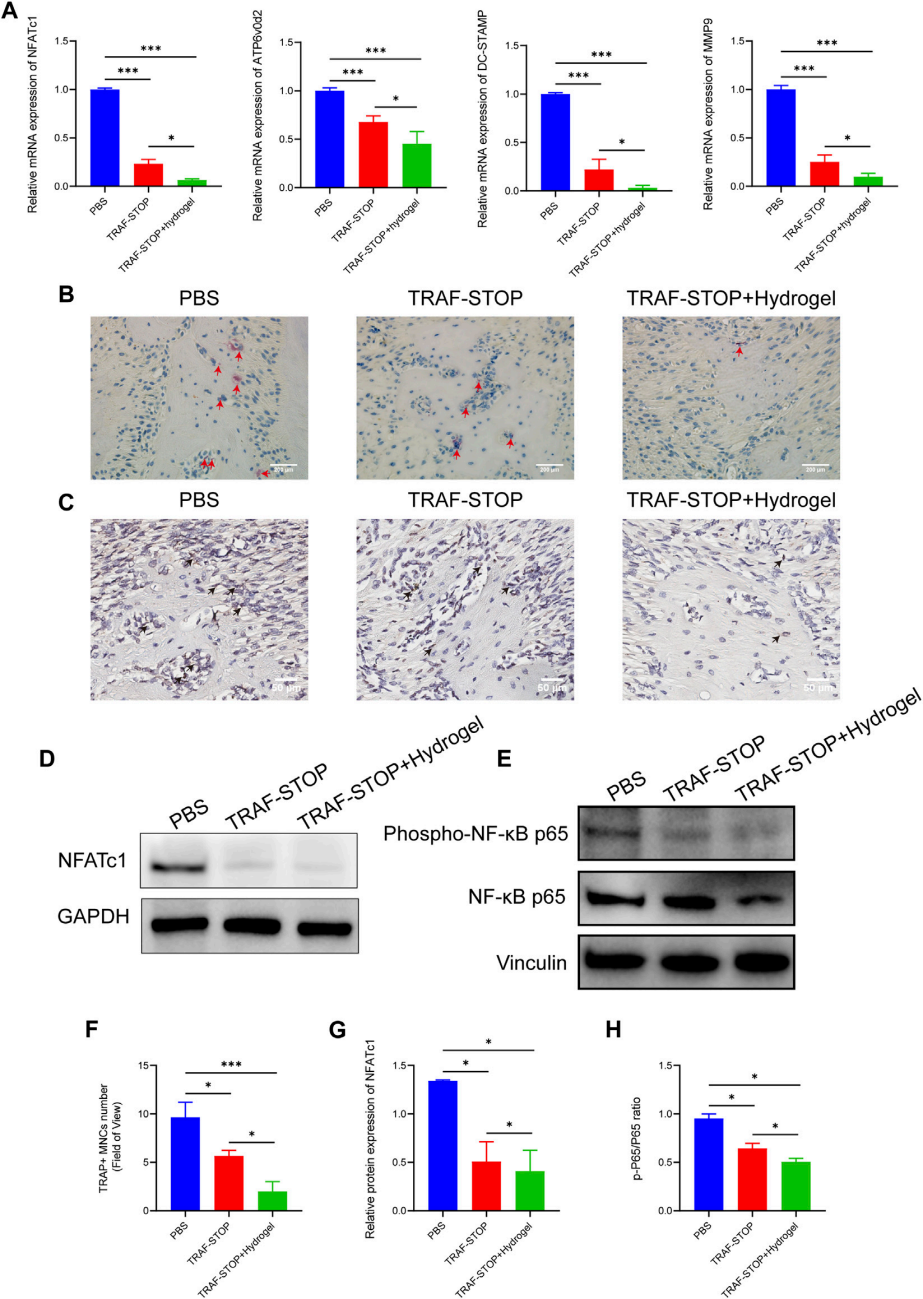
Local administration of TRAF-STOP/hydrogel prevents bone loss through alleviating osteoclast activity. **(A)** Osteoclast-specific genes NFATc1, ATP6v0d2, DC-STAMP, and MMP9 were analyzed using quantitative PCR. All target genes were normalized to the expression of GAPDH mRNA. **(B)** Representative TRAP-stained maxillae images of each group (*n* = 5). Osteoclasts are stained red. Scale bar = 200 μm. **(C)** Representative IHC images of NFATc1 expression from each group (*n* = 5). Scale bar = 50 μm. **(D)** The NFATc1 protein expression levels of each group were presented by Western blotting (*n* = 5). **(E)** The total and phosphorylated P65 protein expressions levels of each group were presented by Western blotting (*n* = 5) **(F)** Statistical analysis of the TRAP-stained cells in each group (*n* = 5). **(G)** Quantitative analysis of NFACT1 normalized to GAPDH expression. **(H)** Relative protein expression of p-P65/P65 was quantified by ImageJ software. (Data are expressed as mean ± SD. *p < 0.05, **p < 0.01, ***p < 0.001.).

The reduction of osteoclastogenesis was confirmed by qRT-PCR analysis (Figure 6A). Both experimental groups exhibited a lower expression of osteoclast genes encoding NFATc1, ATP6v0d2, DC-STAMP, and MMP9 compared to the control group, with the greatest reduction in TRAF-STOP/hydrogel. Later, TRAP staining was performed to investigate whether TRAF-STOP/hydrogel prevents ligature-induced bone loss through the inhibition of osteoclastogenic activity *in vivo* (Figures 6B, F). The number of TRAP-positive multinucleated cells was significantly decreased in TRAF-STOP-injected mice compared to the PBS-injected group, from 9.67 per field of view to 5.67 per field of view. Meanwhile, the numbers of osteoclasts in the periodontium (average of 2 per field of view) were fewest in the TRAF-STOP/hydrogel-treated group. Moreover, the histochemical analysis demonstrated that the expression of NFATc1, the key transcriptional regulator involved in osteoclastogenesis, was distinctly downregulated by TRAF-STOP, and the addition of hydrogel amplified the effect (Figure 6C). In addition, the expression level of the osteoclast marker protein NFATc1 detected by Western blotting was consistent with the above observations, with a statistically 2.6-fold decrease in the TRAF-STOP group and a 3.3-fold reduction in the TRAF-STOP/hydrogel group compared to the PBS group (Figures 6D, G). Furthermore, Western blotting results revealed that the phosphorylation of P65 was visibly inhibited upon exposure to TRAF-STOP and further diminished in the TRAF-STOP/hydrogel group, suggesting that the osteoprotective effect of TRAF-STOP/hydrogel is attributed to the inhibition of NF-κB-mediated osteoclast formation and development (Figures 6E, H).

## 4 Discussion

In this study, we found the potential role of the CD40L-CD40-TRAF6 axis in periodontitis and further confirmed its role in promoting osteoclast activity. Importantly, TRAF-STOP suppressed NF-κB -mediated osteoclast differentiation and the bone resorption capacity of osteoclasts. We also discovered that TRAF-STOP-loaded hydrogel reduced alveolar bone loss and alleviated periodontal inflammation in experimental mice via inhibition of NF-κB-mediated osteoclastogenesis.

CD40 was believed to be a protein exclusive to B cells initially (Paulie et al., 1985; Ledbetter et al., 1987). Indeed, several studies have revealed a considerably wider expression of CD40, which can be detected in epithelial cells, monocytes/macrophages, fibroblasts, and other cells (Schönbeck and Libby, 2001a; Schönbeck and Libby, 2001b). This was well illustrated by the expression of CD40 in gingival immunofluorescence assays. CD40L-CD40 dyad is associated with pathogenic processes of chronic inflammatory diseases, such as rheumatoid arthritis, atherosclerosis, and cardiovascular disease, all of which have been linked to the development of periodontitis (Hajishengallis, 2015). Given that periodontitis and the above-mentioned diseases share similar pathogenic pathways, we hypothesize that there may be a link between CD40L-CD40 and periodontitis. Mahanonda et al. (2004) discovered the expression of CD40 on Monocyte was dramatically upregulated by LPS derived from Porphyromonas gingivalis, which is the major pathogenic bacterium of periodontitis (Mahanonda et al., 2004; Gao et al., 2018). Qin et al. (2005) also showed that this phenomenon was also present in macrophages and microglia. Consistently, our study demonstrated the elevated expression of CD40 in periodontitis. Interestingly, Conley et al. pointed out that neither CD40 nor CD40 ligand has any extra ligands or receptors (Conley et al., 2009). Thus, that is, why our experiment did not focus on CD40L expression. Taken together, our findings suggest a role for CD40L-CD40-TRAF6 axis in periodontitis.

It has been demonstrated that the CD40L-CD40 axis played a part in osteoclastogenesis. First, Durie et al. discovered that an antibody to CD40L significantly decreased bone degradation in arthritis, which inspired relevant studies to go further (Durie et al., 1993). Later, Li et al. (2011) performed relative experiments on ovariectomy mice, a standard model for the onset and progression of osteoporosis brought on by menopause. However, ovariectomy failed to cause bone loss in CD40L−/− mice, and it was verified that ovariectomy increased osteoclast activity through CD40L, indicating the significance of the CD40L-CD40 axis to osteoclastogenesis. Furthermore, Bishop et al. confirmed the active role of CD40L in osteoclast formation and inventively applied TRAF-STOP to diseases associated with osteoclasts *in vivo*, revealing that TRAF-STOP could inhibit bone resorption caused by breast cancer (Bishop et al., 2020). In line with earlier findings, the addition of CD40L in our experiment stimulated the CD40L-CD40-TRAF6 axis, contributing to the activation of osteoclasts, which was reversed by TRAF-STOP both *in vivo* and *in vitro*. However, Kadono et al. (2005) reported that the CD40 stimulated by antibodies or CD40L did not enhance the RANKL-induced osteoclastogenesis. The contradictory result may be attributed to the variety and concentration of agonists to CD40L.

Interestingly, we noted that the addition of CD40L alone did not lead to osteoclast formation in the absence of RANKL (Supplementary Figure S2) despite CD40 and RANK sharing a distinctive TRAF6-binding motif (Marino et al., 2022). Consistently, Kadono et al. (2005) discovered CD40 stimulated by CD40L did not induce osteoclast differentiation of BMMs, which was further convinced by Gohda et al. (2005) through the extracellular domain analysis (Gohda et al., 2005; Kadono et al., 2005). They pointed out that the potential to promote osteoclastogenesis was determined not by the quantity of TRAF6-binding sites but by the capacity of future NFATc1 induction, in which RANKL -TRAF6 is theoretically more potent. NFATc1 is a major regulator of osteoclastogenesis, which is activated and automatically amplified by sustained Ca^2+^ oscillations (Kang et al., 2020). Once activated, NFATc1 dephosphorylates and undergoes nuclear translocation, inducing osteoclast-specific gene expression in the nucleus and promoting osteoclast differentiation (Negishi-Koga and Takayanagi, 2009b). Our experiment showed that CD40L facilitated NFATc1 expression and nuclear translocalization when the cells were cultured with RANKL. Thus, to be more precise, the CD40L-CD40-TRAF6 axis plays a regulatory role in the osteoclastic activity determined by the RANKL-RANK-TRAF6 pathway.

As indicated by the synthesizer (Zarzycka et al., 2015), whether TRAF-STOP obstructing the other receptor–TRAF interactions remains unknown. CD40 and RANK are believed to share a distinctive TRAF6 binding modify (Ye et al., 2002). Thus, TRAF-STOP is likely to react with both CD40-TRAF6 and RANK-TRAF6, which was confirmed by the TRAP staining results (Figure 2G). The inhibitory effect of TRAF-STOP does not act exclusively through CD40L-CD40-TRAF6 axis. TRAF-STOP has the potential effect on RANK-TRAF6 contact too. At present we can only be sure that this small molecular inhibitor suppresses osteoclastogenesis. More research is needed to explore the detailed mechanism of TRAF-STOP-mediated osteoclast inhibition. Noteworthily, it would be more appropriate to regard TRAF-STOP as the TRAF6-specific inhibitor to a certain extent. Given the prominent role of TRAF6 in osteoclastogenesis (Lomaga et al., 1999; Walsh et al., 2015), TRAF-STOP may exhibit extraordinary talent in osteoclast-related diseases.

Generally speaking, the activation of the RANKL-RANK-TRAF6 signaling initiates intracellular signaling pathways, of which NF-κB is the critical one for osteoclast differentiation (Jimi et al., 2004). Bone sclerosis develops in mice that lack the NF-kappaB1 and NF-kappaB2 proteins due to abnormalities in osteoclast differentiation (Iotsova et al., 1997). In addition, activation of NF-κB signaling is often observed in patients with periodontitis (Kurgan and Kantarci, 2018). Some researchers regarded NF-κB as a key target for the therapy of bone loss brought on by inflammatory illnesses like periodontitis (Jimi and Ghosh, 2005). Kure et al. (2019) found that blocking NF-κB signaling pathway by IkB kinase inhibitor effectively alleviated ligature-induced periodontitis in mice (Kure et al., 2019). In our study, TRAF-STOP could efficiently block the NF-κB pathway in both RANKL-induced BMM cells and experimental periodontitis mice, which may account for its inhibitory effect on osteoclastogenesis. On the other hand, the inhibition of the NF-κB pathway further proved its value in lowering periodontal inflammation. Based on the driving effect of NF-κB pathway on pro-inflammatory cytokines, the downregulation of IL-1β, IL-6 and TNF-α is likely to be attributed to inhibition of NF-κB pathway (Chen and Greene, 2004). Moreover, the dual-sided therapeutic action raises the possibility that the NF-κB pathway regulates the interaction between bone metabolism and immune responses (Jimi and Ghosh, 2005).

Both PEG and PLGA meet FDA approval for pharmaceutical applications and the copolymers are harmless when hydrolyzed (Zhang et al., 2014). The loaded medicine is continually delivered to the target by PLGA-PEG-PLGA hydrogel, which is helpful to raise the drug concentration around the periodontal lesion and decrease the frequency of local administration (Wang et al., 2020). Both modified hydrogels and hydrogels loaded with therapeutic effecting molecules have achieved positive results in periodontal therapy (Xu et al., 2019; Shen et al., 2020; Guo et al., 2021). Our study demonstrates local injection of TRAF-STOP-loaded PLGA-PEG-PLGA hydrogel effectively alleviated ligation-induced periodontitis. TRAF-STOP’s hydrophobicity tends to reduce its bioavailability and restrict its medicinal efficacy. The hydrogel-driven controlled release of the drug can maximize bioavailability and showed better therapeutic results as desired (Zhao et al., 2017; Bojadzic and Buchwald, 2018). Thus, PLGA-PEG-PLGA hydrogel is expected to be a promising drug delivery system for the management of bone loss related to clinical periodontitis.

## Data Availability

The original contributions presented in the study are included in the article/Supplementary Material, further inquiries can be directed to the corresponding authors.
