# Supplementary material for: TRAF-STOP alleviates osteoclastogenesis in periodontitis
Source: Front Pharmacol. 2023 May 15;14:1119847. doi: 10.3389/fphar.2023.1119847 (PMC10229065; doi:10.3389/fphar.2023.1119847)
Supplement: Supplementary file 1 [file Table1.DOCX]

Supplementary Materials

TRAF-STOP alleviates osteoclastogenesis by blocking the CD40L-CD40-TRAF6 pathway in periodontitis

**Yaxian Huang^1†^, Jinyan Wu^1†^, Chi Zhan^1†^, Rong Liu^2†^, Zhaocai Zhou^1^, Xin Huang^1^, Yaguang Tian^3^, Zhengmei Lin^1*^, Zhi Song^1*^**

*** Correspondence:**

Zhengmei Lin, Hospital of Stomatology, Guangdong Provincial Key Laboratory of Stomatology, Guanghua School of Stomatology, Sun Yat-sen University, Guangzhou 510055, Guangdong, China. Electronic address: [linzhm@mail.sysu.edu.cn](mailto:linzhm@mail.sysu.edu.cn).

Zhi Song, Hospital of Stomatology, Guangdong Provincial Key Laboratory of Stomatology, Guanghua School of Stomatology, Sun Yat-sen University, Guangzhou 510055, Guangdong, China.

Electronic address: [songzh@mail.sysu.edu.cn](mailto:songzh@mail.sysu.edu.cn).

# Supplementary Figures and Tables

**Supplementary Figure 1**

**
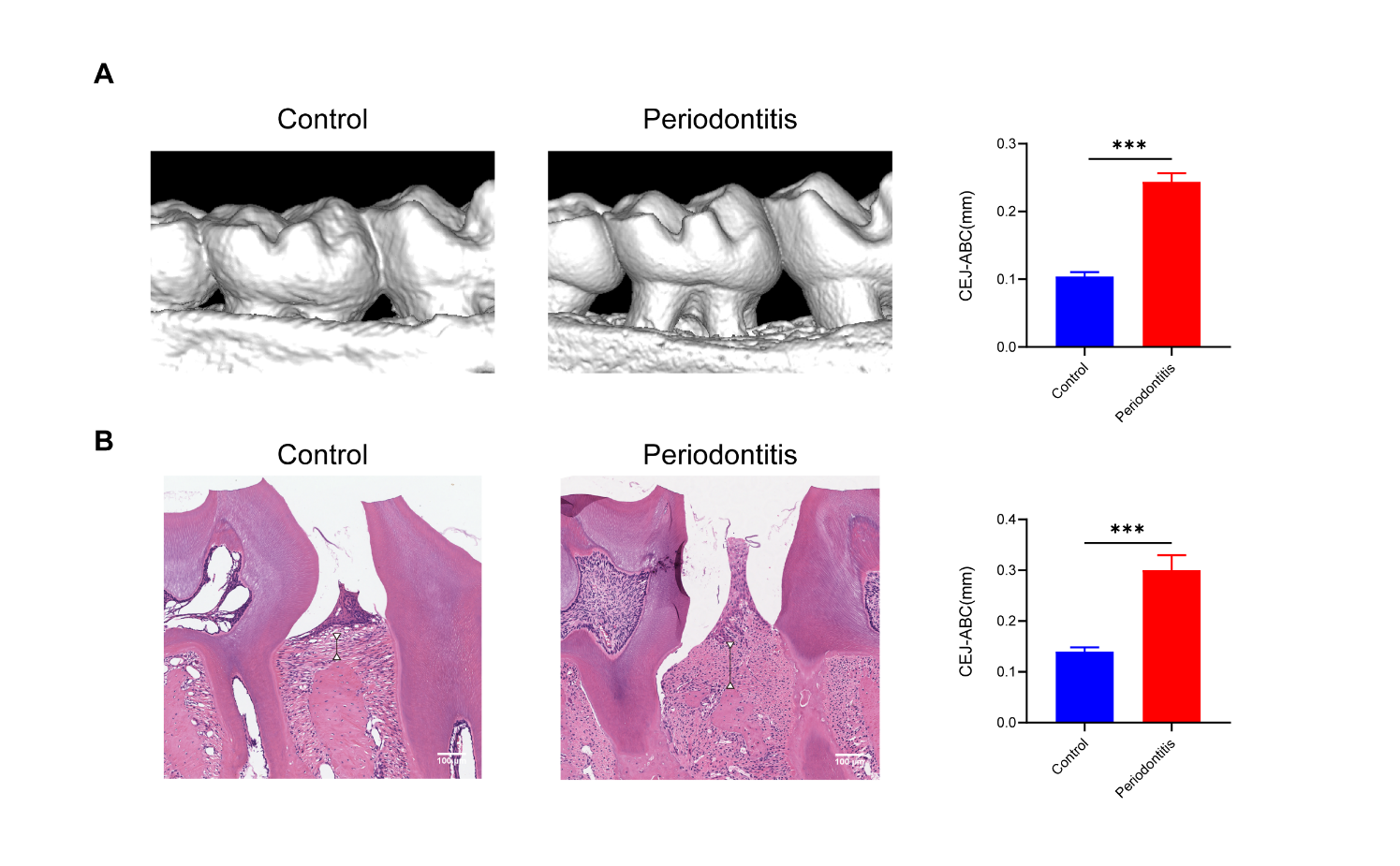
**

**Supplementary Figure 1.** Periodontitis model was successfully established

1. Representative images of 3D reconstructions of maxillae from each group and statistical analysis of the Micro-CT-based CEJ-ABC distance (*n* = 5). The vertical line indicates the distance between CEJ-ABC.
2. Representative histological images of H&E -stained maxillae sections in various groups and statistical analysis of the H&E staining-based CEJ-ABC distance (*n* = 5). The vertical line exhibits the distance between CEJ to ABC. Scale bar = 100 μm.

**Supplementary Figure 2**


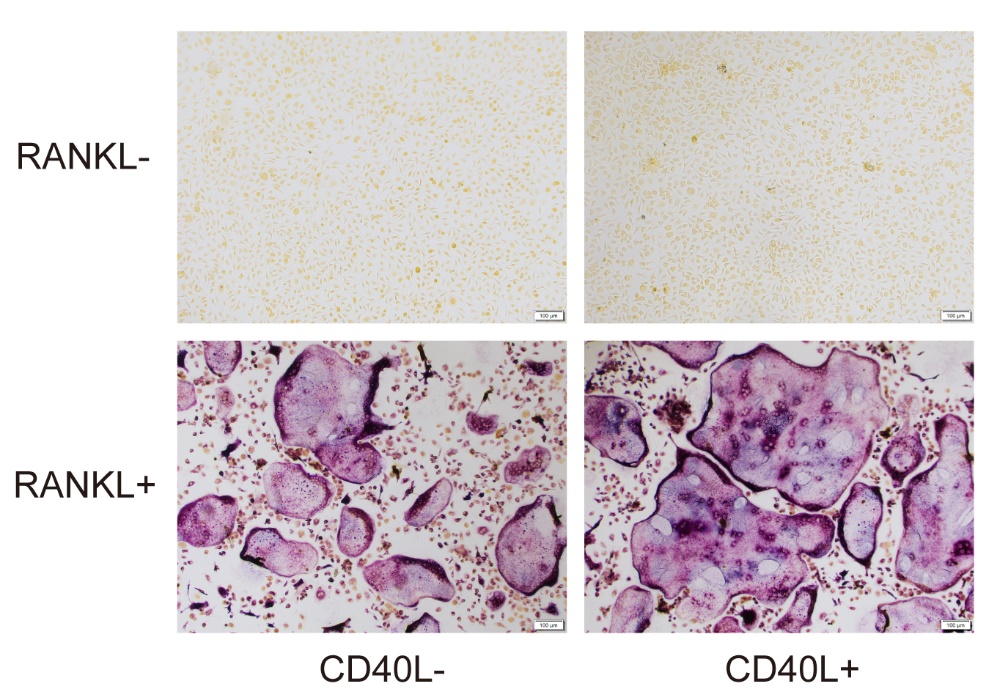


**Supplementary Figure 2.** Representative images of TRAP-stained multinucleated osteoclasts treated with or without CD40L in the absence or presence of RANKL. Scale bar = 100 μm.

**Supplementary Table 1. Primers used in this study**

| GENE | PRIMER SEQUENCE (5'-3'） |
| --- | --- |
| GENE for human  CD40-F | CCTCTTGGTGCTGGTCTTTATC |
| CD40-R | ATGTAAAGTCTCCTGCACTGG |
| TRAF6-F | CACCTACAAGAGAACACCCAG |
| TRAF6-R | AGTCGGGTATAACGCTCAAAC |
| IL-1β-F | TCTTCGACACATGGGATAACG |
| IL-1β-R | GAGGTGGAGAGCTTTCAGTTC |
| IL-6-F | TCTCCACAAGCGCCTTCG |
| IL-6-R | CTCAGGGCTGAGATGCCG |
| TNF-α-F | CTCACCCACACCATCAGC |
| TNF-α-R | GAAGACCCCTCCCAGATAGA |
| GADPH-F | AATCCCATCACCATCTTCCAG |
| GAPDH-R | AAATGAGCCCCAGCCTTC |
| GENE for mice |  |
| NFATc1-F | TGGGAGATGGAAGCAAAGAC |
| NFATc1-R | ATAGAAACTGACTTGGACGGG |
| c-Fos-F | CCAGTCAAGAGCATCAGCAA |
| c-Fos-R | AAGTAGTGCAGCCCGGAGTA |
| TRAP-F | GATGACTTTGCCAGTCAGCA |
| TRAP-R | AACTGCTTTTTGAGCCAGGA |
| MMP9-F | TCCAGTACCAAGACAAAGCCTA |
| MMP9-R | TTGCACTGCACGGTTGAA |
| ATP6v0d2-F | GACCCTGTGGCACTTTTTGTATTC |
| ATP6v0d2-R | GCTTGCATTTGGGGAATCTATC |
| DC-STAMP-F | AAAACCCTTGGGCTGTTCTT |
| DC-STAMP-R | AATCATGGACGACTCCTTGG |
| IL-1β-F | ACGGACCCCAAAAGATGAAG |
| IL-1β-R | TTCTCCACAGCCACAATGAG |
| IL-6-F | AAACCGCTATGAAGTTCCTCTC |
| IL-6-R | GTGGTATCCTCTGTGAAGTCTC |
| TNF-α-F | ACCCTCACACTCACAAACC |
| TNF-α-R | CTGGGAGTAGACAAGGTACAAC |
| GAPDH-F | CCACTCACGGCAAATTCAAC |
| GAPDH-R | CTCCACGACATACTCAGCAC |

**Supplementary Table 2. Antibodies used in WB**

| MARKER (SPECIES) | DILUTION | DISTRIBUTOR/SOURCE  (CATALOGUE NUMBER) |
| --- | --- | --- |
| Primary antibodies: |  |  |
| CD40 Rabbit mAb | 1:1000 | ABclonal (A0218) |
| TRAF6 Rabbit mAb | 1:1000 | ZEN-BIOSCIENCE (380803) |
| Phospho-NF-κB p65 Rabbit mAb | 1:500 | Affinity( AF2006) |
| NF-κB p65 Rabbit mAb | 1:1000 | ZEN-BIOSCIENCE (380172) |
| NFATc1 Rabbit mAb | 1:1000 | ABclonal (A1539) |
| IL-1β Rabbit mAb | 1:1000 | ABclonal ( A11369) |
| GAPDH Rabbit mAb | 1:3000 | ZEN-BIOSCIENCE (R24404) |
| Secondary antibodies: |  |  |
| Anti-rabbit IgG HRP-linked Ab | 1:5000 | CST (7076) |
